# Association between Platelet Count with 1-year Survival in Patients with Cancer Cachexia

**DOI:** 10.7150/jca.62788

**Published:** 2021-10-30

**Authors:** Yuying Liu, Yizhong Ge, Qinqin Li, Guotian Ruan, Qi Zhang, Xi Zhang, Meng Tang, Mengmeng Song, Xiaowei Zhang, Xiangrui Li, Kangping Zhang, Ming Yang, Chunlei Hu, Tong Liu, Hailun Xie, Yongbing Chen, Kaiying Yu, Minghua Cong, Wei Li, Zhengping Wang, Hanping Shi

**Affiliations:** 1 Institute of Biopharmaceutical Research, Liaocheng University, Liaocheng, Shandong 252000, China.; 2 Department of Gastrointestinal Surgery/Department of Clinical Nutrition, Beijing Shijitan Hospital, Capital Medical University, Beijing, 100038, China.; 3 Beijing International Science and Technology Cooperation Base for Cancer Metabolism and Nutrition, Beijing, 100038, China.; 4 The Second Affiliated Hospital and Yuying Children's Hospital of Wenzhou Medical University, Wenzhou, 325000, China.; 5 Comprehensive Oncology Department, National Cancer Center/Cancer Hospital, Chinese Academy of Medical Sciences and Peking Union Medical College, Beijing, 100038, China.; 6 Cancer Center, the First Hospital, Jilin University, Changchun, 130021, China.

**Keywords:** platelet count, survival, cancer cachexia, nested case-control study

## Abstract

**Background:** Changes in platelet count (PLT) are strongly associated with patient survival and may be clinically indicative of certain underlying diseases. However, there were few studies on the prognosis of patients with cancer cachexia.

**Objective:** The purpose of this study was to investigate the relationship between PLT and 1-year survival in patients with cancer cachexia.

**Methods:** We performed a nested case-control study of data from a multicenter clinical study of cancer. There were 252 patients with cancer cachexia whose survival time was less than or equal to 1 year and 252 patients with cancer cachexia whose survival time was more than 1 year meeting the inclusion criteria. The mortality risk and the adjusted risk were estimated by logistic regression and displayed as odds ratios (ORs) and 95% confidence intervals (95% CIs).

**Results:** PLT was negatively correlated with 1-year overall survival (OS) of patients with cancer cachexia (increased per standard deviation (SD): OR = 1.29; 95% CI: 1.05-1.60;* P* = 0.018). The higher the PLT, the lower the OS of patients. When classified by dichotomy (D1 < 296×10^9^/L, D2 ≥ 296×10^9^/L), OS of patients in the D2 group was worse (OR = 2.18; 95% CI: 1.38-3.47; *P* = 0.001). When classified by quartile (Q1- Q3 < 305×10^9^/L, Q4 ≥ 305×10^9^/L), OS of patients in the Q4 group was poorer (OR = 1.82; 95% CI: 1.14-2.94; *P* = 0.013). In addition, patients with a low PLT (< 296×10^9^/L) and either a high total bilirubin (TBIL) (≥ 17.1 µmol/L) or a smoking history had poor 1-year survival. Based on our primary cohort study, we conducted a survival analysis of 3130 patients with cancer cachexia and found that OS was better in patients with low PLT (< 296×10^9^/L).

**Conclusion:** PLT was negatively correlated with 1-year overall survival of patients with cancer cachexia.

## Introduction

Cachexia is extremely common among all cancer deaths worldwide, with a prevalence of more than 50% 1-3. Its incidence varies according to the type of tumor and is relatively high in gastric and pancreatic cancer (approximately 80%) but relatively low in breast cancer and leukemia (approximately 40%) 2. In 2011, the International Delphi Consensus Process defined cancer cachexia as a multifactorial syndrome of sustained muscle loss (with or without adipose loss) that cannot be completely reversed by conventional nutritional support and leads to progressive functional impairments 4. Cachexia patients often develop the following clinical manifestations: anorexia (or decreased food intake), enhanced catabolic metabolism, decreased muscle mass and strength, social and psychological disorders, and even death 5. Therefore, how to intervene in the development of cachexia as well as improve the patients' quality of life has become an urgent issue in clinic.

In recent years, the relationship between platelets and cancer has received extensive attention. Platelet count (PLT) is associated with prognosis in many diseases. For instance, it can be used as a predictor of death and graft loss after liver transplantation. Patients with a PLT < 70 × 10^9^/L on the fifth day following liver transplantation presented a high mortality rate and poor graft survival within one year after operation 6, and it had also been reported that decreased PLT level was significantly associated with increased total risk of death 7. In addition, similar results were noted by the Women's Health Initiative (limited to post-menopausal females), in which low and high deviations from baseline and average platelet counts were positively correlated with total mortality, coronary heart disease (CHD) mortality, cancer mortality, and non-CHD/non-cancer mortality 8. Furthermore, PLT plays a critical role in several steps of tumor development, including but not limited to tumor growth, angiogenesis, and metastasis of malignancies 9.

This study aims to explore the predictive function of PLT in the clinic, since in addition to its crucial role in hemostasis, platelet is increasingly recognized as an inflammatory mediator regulating the immuno-oncological system 10. It was worth noting that it may play a critical predictive role in clinical practice, as several studies have reported the association between PLT and cancer prognosis 11-14. However, few prospectively prognostic studies of PLT in the cachectic population are currently available. In view of this, this study aimed to investigate the association between PLT and overall survival (OS) in patients with cancer induced cachexia.

## Materials and Methods

### Participants

**Figure 1:**
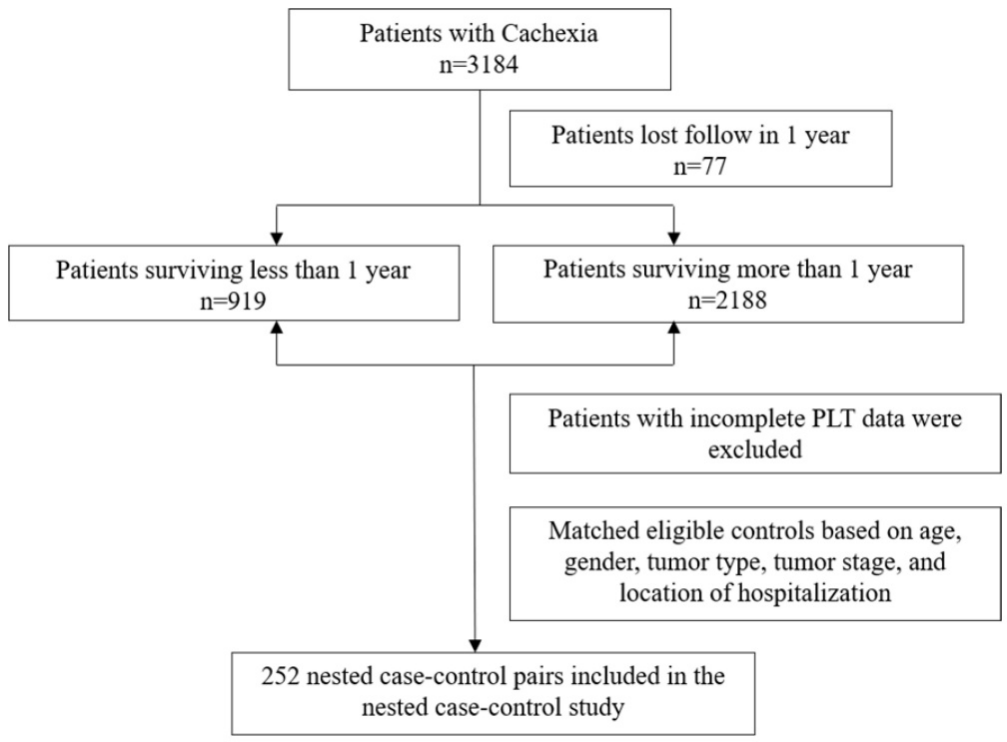
Flowchart of the study participants.

This study was a nested case-control study with data obtained from 40 clinical centers in China from 2013 to 2020. Cancer patients aged 18 years or older were enrolled and patients with incomplete PLT data were excluded (Figure 1). Currently, this study has been approved by the Medical Ethics Review Committee of the registered hospital (Beijing Shijitan Hospital) and conducted in accordance with the *Declaration of Helsinki*. In total, we identified 252 patients with cancer cachexia whose survival time was less than or equal to 1 year and matched 252 controls with a survival time of more than 1 year. We then paired the case and control groups in a 1: 1 ratio based on age (±5 years), gender, tumor type, tumor stage, and location of hospitalization. The median survival estimates along with the two-sided 95% CI of patients with survival time greater than 1 year and patients with survival time less than or equal to 1 year were as follows: 37.5 months (95%CI, 27.8 to 36.2) and 6.13 months (95%CI, 5.50 to 6.80), respectively. All pathological stages in our study were defined in accordance with the American Joint Committee on Cancer TNM staging system (8^th^ edition) 15.

### Diagnosis of Cancer Cachexia and Evaluation of Anthropometric and Lifestyle Factors

The diagnosis of cancer cachexia was based on Fearon's criteria 4. Body mass index (BMI) was calculated as follows: BMI (kg/m^2^) = weight (kg) / height^2^ (m^2^). Mid-upper arm circumference (MAC) and triceps skin fold (TSF) were measured at the acromion and at the midpoint of the olecranon crest of the dominant arm. The subject was placed in a supine position with the knee flexed 90 degrees. MAC was measured with a plastic metric tape, while TSF was measured with a conventional skin crease caliper. A Jamar dynamometer was employed to measure hand grip strength (HGS) of the dominant hand. Information on smoking status, alcohol consumption and tea consumption were obtained through a lifestyle questionnaire. The OS was the primary outcome in this study, which included mortality due to any cause. Evidence of death was obtained from regular follow-up of the patients.

### Laboratory Analysis

The subjects of laboratory testing mainly included total protein, albumin, neutrophils, total bilirubin (TBIL), aspartic transaminase (AST), alanine transaminase (ALT), hemoglobin, white blood cell (WBC), lymphocyte, red blood cell (RBC), as well as PLT. All blood tests were performed after at least 9 hours of fasting and before anti-tumor treatment within the first 24-hour hospitalization. All the study outcomes were reviewed and adjudicated by an independent Endpoint Adjudication Committee, whose members were unaware of the specific assignments of study group.

### Cohort Study Analysis

Prognostic validation was performed in the cachexia cohort based on the truncation level of the nested case-control study. We collected data on 50,000 patients with cancer from 2013 to the end of 2020, and then divided them into a high PLT (≥ 296×10^9^/L) group and a low PLT (< 296×10^9^/L) group. Ultimately, 3130 patients with cancer cachexia were identified based on clinical diagnoses in the medical records, and a subsequent cohort study was conducted according to the matching principle of a 1: 1 ratio.

### Statistical Analysis

Baseline characteristics were represented as means. Differences in categorical variables in baseline characteristics between the case and control groups were compared using the chi-square test, whereas continuous variables were compared using the Wilcoxon rank sum test or the t-test. In this study, odds ratios (ORs) and 95% confidence intervals (95% CIs) for 1-year survival of cancer patients were constructed by modeling risk factors as continuous variables, as well as modeling dichotomous and quartile PLTs using the chi-square test. Adjusted matching variables included BMI, HGS, MAC, TBIL, WBC, RBC, TSF, chemotherapy, radiotherapy, and surgery. Correction factors were selected using stepwise regression. In addition, heterogeneity among subgroups was evaluated by a conditional logistic regression method, and the influence of preoperative treatment was excluded by sensitivity analysis, and the interaction between PLT and subgroups was examined by probability ratio. Survival analysis of the basic cohort (n = 3130) was performed by the Kaplan-Meier method and survival curves. In this study, a two-tailed *P* < 0.05 was considered statistically significant. All analyses were performed by the R software, version 4.0.2.

## Results

### Characteristics of Patients

Compared with patients who survived more than 1 year, patients who survived less than or equal to 1 year had higher levels of WBC (7.86×10^9^/L ± 3.98×10^9^/L vs. 6.45×10^9^/L ± 3.21×10^9^/L), neutrophil count (5.59×10^9^/L ± 3.77×10^9^/L vs. 4.36×10^9^/L ± 3.90×10^9^/L), as well as PLT (261×10^9^/L ± 116×10^9^/L vs. 238×10^9^/L ± 96×10^9^/L). However, HGS (21.85 kg ± 9.56 kg vs. 24.25 kg ± 8.50 kg), BMI(20.15 kg/m^2^ ± 2.90 kg/m^2^ vs. 21.18 kg/m^2^ ± 3.26 kg/m^2^) total protein (65.28 g/L ± 8.47 g/L vs. 67.63 g/L ± 6.86 g/L) and albumin (35.09 g/L ± 5.32 g/L vs. 38.30 g/L ± 5.92 g/L) levels, blood components including Hb (113.29 g/L ± 20.76 g/L vs. 120.38 g/L ± 17.97 g/L), lymphocyte count (1.33×10^9^/L ± 0.76×10^9^/L vs. 1.53×10^9^/L ± 0.65×10^9^/L), RBC (3.90×10^12^/L ± 0.72×10^12^/L vs. 4.16×10^12^/L ± 0.59×10^12^/L), as well as MAC (24.44 cm ± 3.29 cm vs. 25.22 cm ± 3.66 cm) and TSF (12.27 mm ± 6.83 mm vs. 14.53 mm ± 7.37 mm) were lower in the patient population with shorter survival (Table 1). The above variables were used as adjustment variables for the case-control matching analysis.

### The Relationship between PLT and 1-year OS of the Patients with Cancer Cachexia

**Figure 2:**
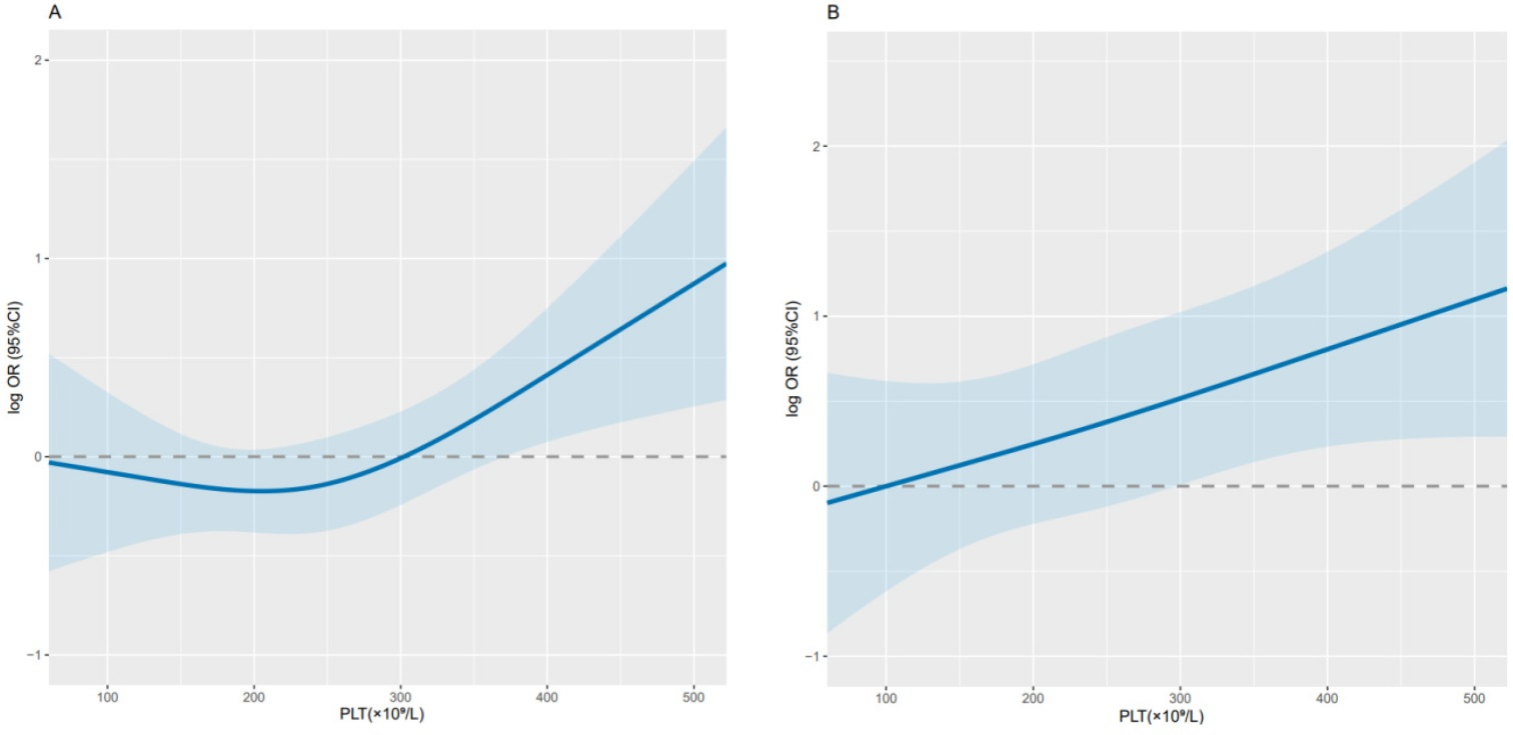
** Relationship between PLT and 1-year survival in patients with cancer cachexia.** Notes: Use conditional logistic regression to analyze the data before (A; per SD increment-*P*=0.015; OR=1.23; 95% CI: 1.04-1.46) and after adjustment (B; per SD increment-*P*=0.018; OR=1.29; 95% CI: 1.05-1.60). Adjusted for BMI, HGS, MAC, albumin, TBIL, WBC, RBC, TSF, chemotherapy, radiotherapy, surgery. PLT: platelet count; CI: confidence interval; OR: odds ratio; BMI: body mass index; HGS: hand grip strength; MAC: mid-upper arm circumference; TBIL: total bilirubin; WBC: white blood cell; RBC: red blood cell; TSF: triceps skin fold.

Overall, PLT was significantly correlated with 1-year survival in cancer cachexia patients (per SD increment-OR = 1.29; 95% CI: 1.05-1.60) (Table 2). The adjusted curve showed a linear trend, suggesting that the higher the PLT, the lower the OS of patients (Figure 2). When dichotomizing PLT (D1 < 296×10^9^/L, D2 ≥ 296×10^9^/L), we found that the D2 group had poorer OS compared with the D1 group (adjusted OR = 2.18; 95 % CI: 1.38-3.47; adjusted *P* = 0.001). While when patients' PLT levels were divided into quartiles (Q1-Q3 < 305×10^9^/L, Q4 ≥ 305×10^9^/L), the Q4 group had a relatively higher risk (adjusted OR = 1.82; 95% CI: 1.14-2.94; adjusted *P* = 0.013) and worse 1-year OS compared with the Q1-Q3 group (Table 2). Through sensitivity analysis, we ruled out the effect of radiotherapy and chemotherapy on the results, which was consistent with the initial results (Table 3).

### Subgroup Analyses

**Figure 3:**
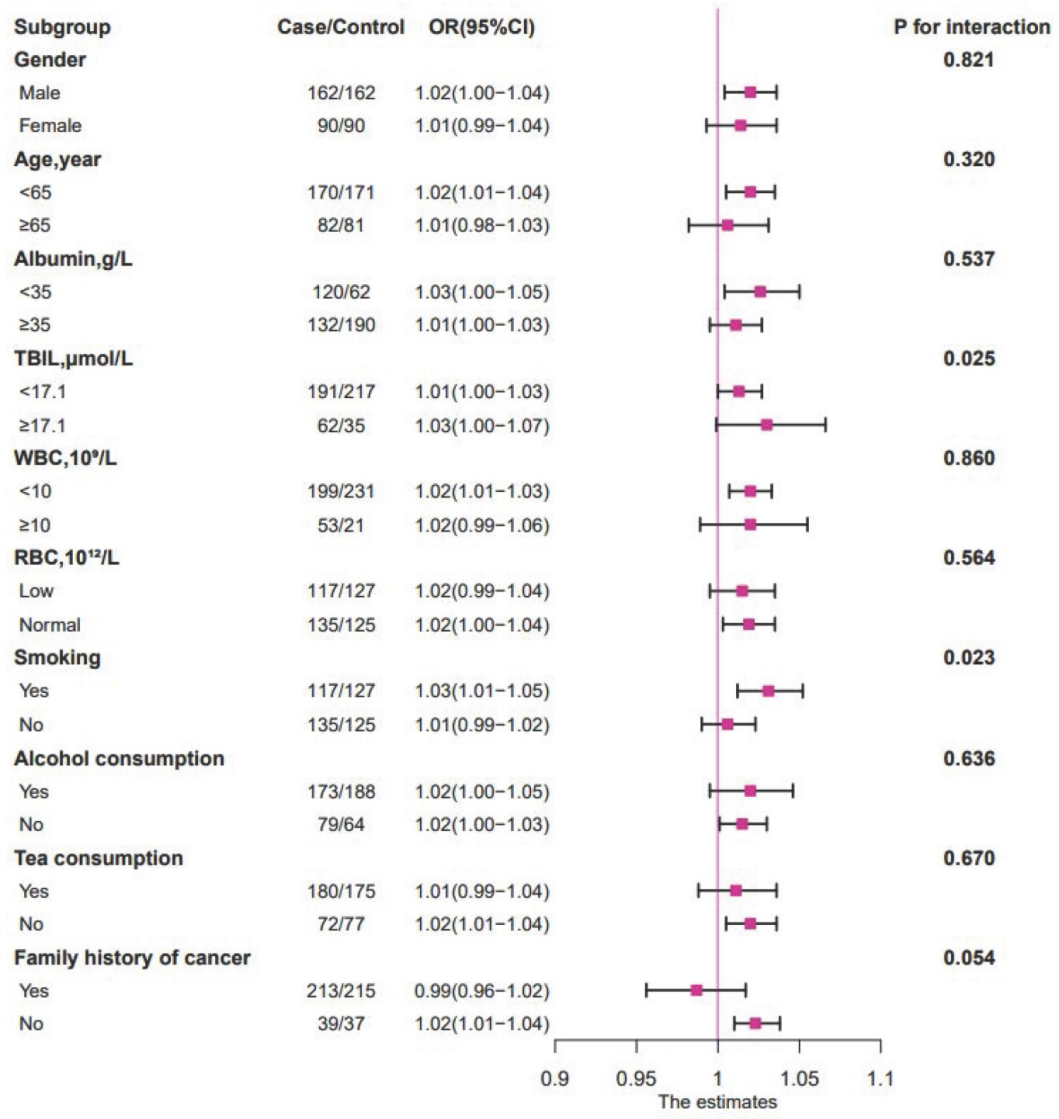
** The relationship between PLT (as continue value) and the 1-year OS of patients with cancer cachexia in different subgroups.** Notes: The conditional logistic regression model was used to calculate the relationship between ORs and PLT (as continue value) of patients with cancer cachexia at 1 year. Each subgroup was adjusted for BMI, HGS, MAC, albumin, TBIL, WBC, RBC, TSF, chemotherapy, radiotherapy, surgery. OR: odds ratio; *P*: probability; TBIL: total bilirubin; WBC: white blood cell; RBC: red blood cell.

**Figure 4:**
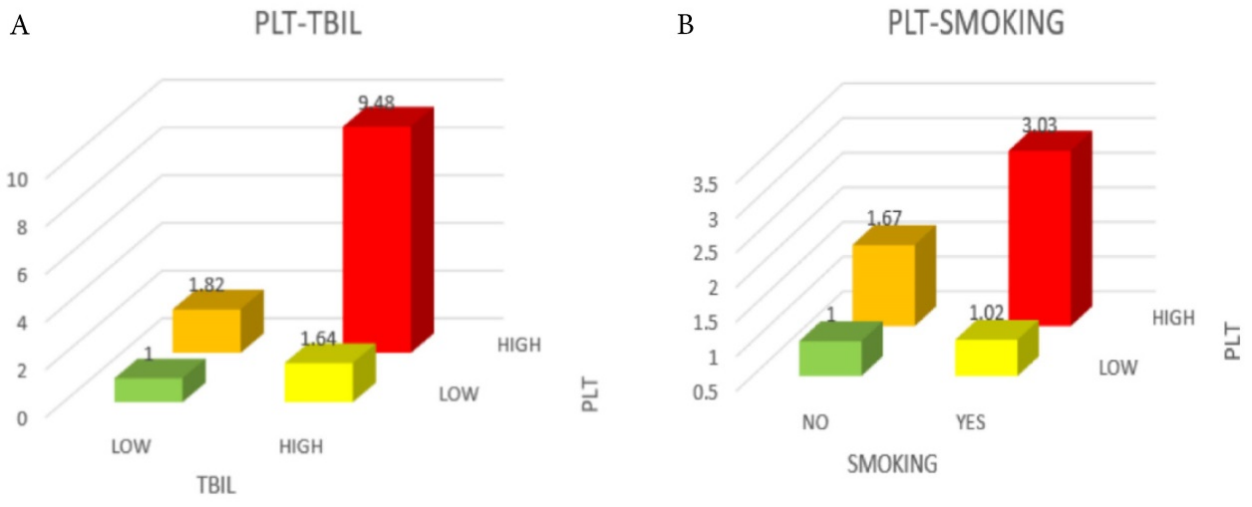
** Comparison of mortality risk among different groups of patients. A,** Green was in the OR range of 0-1, yellow and orange were in the range of 1-2, and red was in the range of 9-10. **B,** Green was in the OR range of 0-1, yellow was in the range of 1-1.5, orange was in the range of 1.5-2, and red was in the range of 3-3.5. Notes: PLT: platelet count; TBIL: total bilirubin; OR: odds ratio.

When the relationship between PLT and survival time was evaluated in different subgroups by stratified analysis (Figure 3), it could be observed that high TBIL level (≥ 17.1 µmol/L), smoking history, and high PLT (≥ 296×10^9^/L) were negatively correlated with patient prognosis (*P* < 0.05). The negative association between PLT and 1-year survival was stronger in the high TBIL group (OR = 1.03; 95% CI: 1.00-1.07; *P* = 0.025) than in the low TBIL group (OR = 1.01; 95% CI: 1.00-1.03) (Figure 3, Figure 4). Similarly, the negative correlation between PLT and 1-year survival was stronger in patients with cancer cachexia (OR = 1.03; 95% CI: 1.01-1.05; *P* = 0.023) than in those without a history of smoking (OR = 1.01; 95% CI: 0.99-1.02) (Figure 3, Figure 4).

### Validation in a Cohort of Patients with Cancer Cachexia

**Figure 5:**
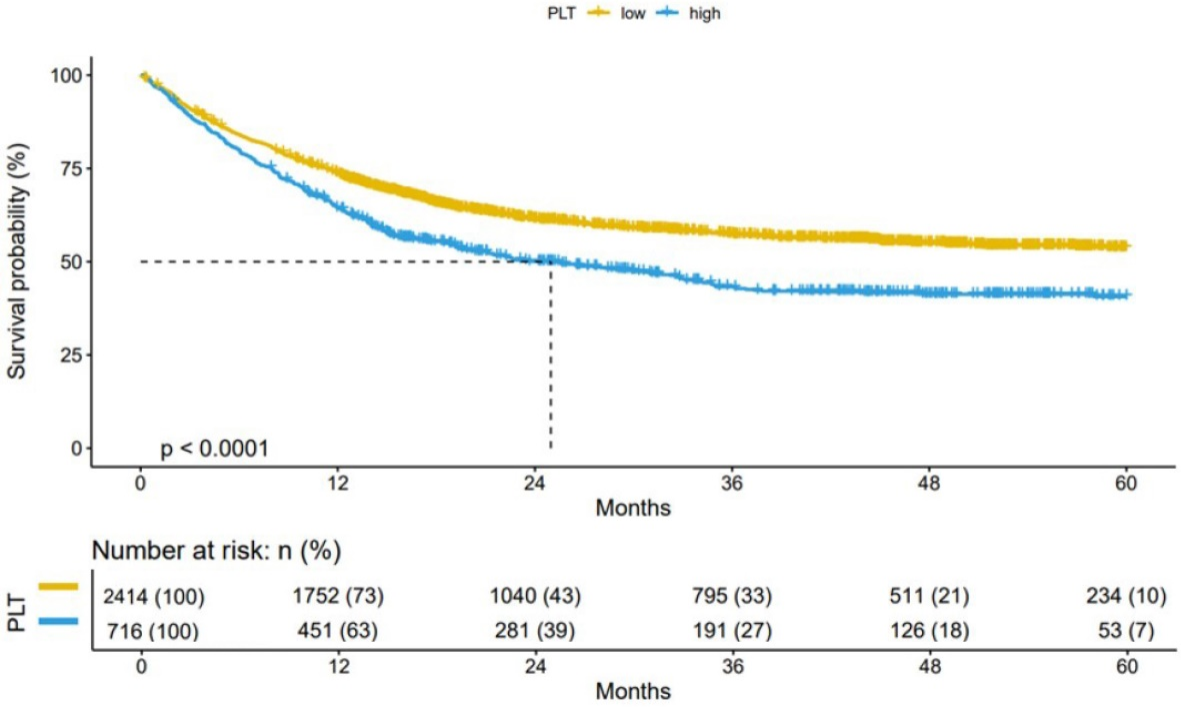
** Results of the Kaplan-Meier survival analysis in PLT-stratified patients with cancer cachexia.** Notes: Patients with cancer cachexia were followed up for more than 1 year (n=3130). PLT: platelet count; *P*: probability.

Based on the cut-off level (296×10^9^/L) of the nested case-control study, we performed prognostic validation in the cachexia cohort. The cohort study (n=3130) showed that compared with patients with high PLT (≥ 296×10^9^/L), patients with low PLT (< 296×10^9^/L) had better OS (Figure 5). Thus, providing validation for the reliability of the established cut-off for PLT in cancer cachexia patients.

## Discussion

Overall, in this hospital-based retrospective nested case-control study, a higher PLT was associated with a poorer OS. The relationship between PLT and survival has been examined in several previous studies. In an analysis of 285 patients with non-small cell lung cancer who underwent consecutive therapeutic pneumonectomy, the rates of thrombocytosis were 22.41% and 3.82% in stage III + IV and stage I patients, respectively (median PLT: 449×10^9^/L vs. 254×10^9^/L;* P* < 0.001), indicating that thrombocytosis was prevalent in patients with non-small cell cancer 12. In addition, it has been reported that elevated PLT (≥ 400×10^9^/L) could predict poor prognosis in lung cancer patients 14. However, the cut-off values of PLT in the above studies were higher than the normal value, demonstrating no contradiction with the results of our study. Similarly, a cohort study conducted by Lu et al. showed that the median OS of hepatocellular carcinoma patients was highest when the platelet count change (ΔPLT) was in the range of '± 20×10^9^/L', while it decreased when the ΔPLT ≤ or ≥ 20×10^9^/L, which is in favor of our findings^13^. Of note, reports on the association between PLT levels within the normal range and patient survival remain scarce.

Analysis of Q1-Q4 groups indicated that elevated PLT was negatively correlated with OS of cancer cachexia patients. Furthermore, we found interactions between PLT and TBIL levels as well as smoking history in subgroup analyses. Specifically, the OR value was highest (OR=8.98) when both PLT and TBIL were high, suggesting that those patients with higher PLT and TBIL would suffer from a higher risk of death compared to those with lower PLT and TBIL.

A study showed that in stage IV colorectal cancer patients, elevated TBIL and DBIL were associated with poorer OS 16. The optimal cut-off value for TBIL was 12.9 μmol/L, slightly lower than that of this study, which may be attributed to different geographic locations and tumor stages. However, in studies on non-metastatic breast cancer 17, gastric cancer 18, as well as stages II and III colorectal cancer (after radical resection) 19, TBIL was positively correlated with survival. But among them the TBIL cut-off values were all lower than that in our study. Further study of the interaction under normal values for PLT is needed. Furthermore, patients with high PLT and smoking history also had a poor survival (OR = 2.95), partially owing to the fact that smoking causes oxidative stress *in vivo* and leads to platelet activation and aggregation. Meanwhile, smoking may activate thrombopoietin which stimulates platelet production 20. It had also been reported that smoking caused a hypercoagulable state of blood, which directly promoted thrombosis 21.

Platelets, and platelet related indicators, including PLT, mean platelet volume (MPV), platelet distribution width (PDW), platelet crit (PCT), and platelet-to-lymphocyte ratio (PLR), are important in the clinical observation of the prognosis of certain cancers. Using a retrospective analysis, Huang 22 and colleagues found that breast cancer patients with a PDW >16.8% had an overall survival rate of 16.8%, which was significantly shorter than that of patients with a PDW ≤ 16.8%, indicating that PDW may be a prognostic marker in breast cancer. It had also been reported that MPV and PDW could be used as the prognostic indicators for benign and malignant endometrial lesions. In the malignant group, MPV was higher than 7.54 while PDW was lower than 37.8, showing the potential of these two indicators in discriminating between benign and malignant endometrial tumors 23. Similarly, patients with a higher baseline MPV had worse progression free survival and overall survival 24, in consistency with the previous conclusion. Moreover, PLR has been demonstrated to be of reference significance in prediction of prognosis across a variety of cancers. A meta-analysis showed a negative correlation between PLR and OS, as a higher PLR increased the risk of mortality from hepatocellular carcinoma (OR = 1.59; 95% CI: 1.42-2.04; *P* < 0.00001) 25.

The ability of PLT in predicting the prognosis of cancer cachexia patients may be related to thromboembolism. In cancer patients, the endogenous ligand podoplanin binds to C-type lectin-like receptor 2 to induce platelet activation, promoting hematological cancer metastasis and cancer associated thrombosis 26. This hypothesis has been confirmed in animal experiments. In addition, Julia and colleagues, in the study of cancer patients with poor prognosis, found that the mortality and the incidence of venous thromboembolism may be enhanced by excessive platelet activation 27. Another mechanism by which massive platelet activation leads to poor prognosis in cancer patients may lie in the release of a large number of factors that modulate tumor microenvironment after platelet activation. These factors may promote the release of angiogenic growth factors from platelet α-granules and contribute to tumor angiogenesis. The release of proinflammatory cytokines helps remodel extracellular matrix and promotes angiogenesis. In addition, platelets promote circulation, extravasation, as well as epithelial mesenchymal transition at metastatic sites, and facilitate malignant cell colonization 10, 28, 29. The risk of thromboembolism is significantly increased 30, and venous thromboembolism is considered as the main cause of death among cancer patients. Studies have proven that early venous embolism was associated with increased mortality in lung cancer patients 31. Other mechanisms still need to be explored.

Currently, this correlation was found for the first time in our study, which provided great help and convenience for the prognostic management of patients with cancer cachexia. However, this paper has several limitations in the following aspects. First, participants' PLT was evaluated only at baseline, so we could not explore the impact of dynamic changes in PLT on the survival of cancer patients. Second, our included sample size (n=252) was not sufficiently representative of all patients with cancer cachexia. Further studies need to expand the sample size to increase credibility. Third, our study subjects were of a single ethnicity, multi-ethnic studies may be conducted in the future to generalize our conclusions. Finally, this study is short of a systematic review addressing multiple platelet indices (MPV, PDW, PCT, etc.) which could be fixed out in future study design. Due to the aforementioned limitations, these findings require further verification in the future.

## Conclusions

In summary, PLT was negatively correlated with 1-year OS in patients with cancer cachexia, which was validated in the total independent population cohort. In addition, patients with a high TBIL and a smoking history had a lower 1-year survival rate. Our findings, to some extent, provide certain guidance for the prognostic management of patients with cancer cachexia.

## Figures and Tables

**Table 1:** Detailed baseline characteristics of the enrolled patients

Characteristic	Total (n = 504)	> 1year (n = 252)	≤ 1year (n = 252)	*P* value
Age, years, n (%)	59.61 (9.49)	59.69 (9.48)	59.52 (9.51)	0.844
**Gender, n (%)**				1.000
Male	324 (64.30)	162 (64.30)	162 (64.30)	
Female	180 (35.70)	90 (35.70)	90 (35.70)	
**Tumor stage, n (%)**				1.000
I	14 (2.80)	7 (2.80)	7 (2.80)	
II	62 (12.30)	31 (12.30)	31 (12.30)	
III	164 (32.50)	82 (32.50)	82 (32.50)	
IV	264 (52.40)	132 (52.40)	132 (52.40)	
Chronic disease (Yes), n (%)	167 (33.10)	89 (35.30)	78 (31.00)	0.344
Family history (Yes), n (%)	76 (15.10)	37 (14.70)	39 (15.50)	0.901
Smoking, n (%)	260 (51.60)	125 (49.60)	135 (53.60)	0.422
Drinking, n (%)	143 (28.40)	64 (25.40)	79 (31.30)	0.167
Tea consumption (Yes), n (%)	149 (29.60)	77 (30.60)	72 (28.60)	0.696
Nutrition support (Yes), n (%)	199 (39.50)	85 (33.70)	114 (45.20)	0.011
Total protein, g/L	66.45 (7.79)	67. 63 (6.86)	65.28 (8.47)	0.001
Albumin, g/L	36.69 (5.84)	38.30 (5.92)	35.09 (5.32)	<0.001
TBIL, median (IQR), g/L	11.00 [8.00, 15.20]	10.55 [8.00, 14.35]	11.20 [8.07, 16.33]	0.058
AST, median (IQR), U/L	22.00 [17.00, 30.02]	21.00 [17.00, 28.38]	22.95 [17.20, 32.00]	0.093
ALT, median (IQR), U/L	18.80 [13.00, 29.38]	18.80 [13.47, 29.00]	18.45 [12.80, 31.15]	0.970
Hb, g/L	116.83 (19.72)	120.38 (17.97)	113.29 (20.76)	<0.001
WBC, 10^9^/L	7.15 (3.68)	6.45 (3.21)	7.86 (3.98)	<0.001
Neutrophil count, 10^9^/L	4.98 (3.88)	4.36 (3.90)	5.59 (3.77)	<0.001
Lymphocyte count, 10^9^/L	1.43 (0.71)	1.53 (0.65)	1.33 (0.76)	0.001
RBC, 10^12^/L	4.03 (0.67)	4.16 (0.59)	3.90 (0.72)	<0.001
PLT, 10^9^/L	249.35 (106.74)	237.52 (95.92)	261.17 (115.56)	0.013
BMI, kg/m^2^	20.66 (3.13)	21.18 (3.26)	20.15 (2.90)	<0.001
HGS, kg	23.05 (9.12)	24.25 (8.50)	21.85 (9.56)	0.003
TSF, mm	13.40 (7.18)	14.53 (7.37)	12.27 (6.83)	<0.001
MAC, cm	24.83 (3.50)	25.22 (3.66)	24.44 (3.29)	0.012
Surgery, n (%)	127.00 (25.20)	67.00 (26.60)	60.00 (23.80)	0.530
Chemotherapy, n (%)	279.00 (55.40)	155.00(61.50)	124.00 (49.20)	0.007
Radiotherapy, n (%)	28.00(5.60)	9.00 (3.60)	19.00 (7.50)	0.080

Notes: Continuous variables were represented by mean ± standard deviations (SDs), among them, TBIL, AST, ALT were represented by median and interquartile range. Categorical variables were represented by numbers and percentages. Differences in baseline characteristics were compared using the chi-square test, t-test (conform to the normal distribution), or Wilcoxon rank sum test (not conform to the normal distribution). TBIL: total bilirubin; AST: aspartic transaminase; ALT: alanine transaminase; Hb: hemoglobin; WBC: white blood cell; RBC: red blood cell; PLT: platelet count; BMI: body mass index; HGS: hand grip strength; TSF: triceps skin fold; MAC: mid-upper arm circumference.

**Table 2:** Conditional logistic regression analysis of anthropometrics and 1-year OS of patients with cancer cachexia

PLT (×10^9^/L)	Cases/Controls	Unadjusted		Adjusted	
		*P* value	OR (95% CI)	*P* value	OR (95% CI)
Per SD		0.015	1.23 (1.04-1.46)	0.018	1.29 (1.05-1.60)
**By cutoff**					
D1 (< 296)	167/200		ref.		ref.
D2 (≥ 296)	85/52	0.001	2.01 (1.34-3.02)	0.001	2.18 (1.38-3.47)
**Interquartile**					
Q1~Q3 (< 305)	175/203		ref.		ref.
Q4 (≥ 305)	77/49	0.006	1.79 (1.19-2.71)	0.013	1.82 (1.14-2.94)

Notes: The 1-year survival ORs of cancer cachexia patients were estimated by modeling PLT as a continuous variable and using conditional logistic regression as the dichotomy and quartile. The analyses were adjusted for BMI, HGS, MAC, albumin, TBIL, WBC, RBC, TSF, chemotherapy, radiotherapy, surgery. PLT: platelet count; CI: confidence interval; OR: odds ratio; *P*: probability; D: dichotomy; Q: quarter; BMI: body mass index; HGS: hand grip strength; MAC: mid-upper arm circumference; TBIL: total bilirubin; WBC: white blood cell; RBC: red blood cell; TSF: triceps skin fold.

**Table 3:** Sensitivity analysis

PLT (×10^9^/L)	Cases/Controls	Unadjusted		Adjusted	
		*P* value	OR (95% CI)	*P* value	OR (95% CI)
Per SD		0.029	1.21(1.02-1.44)	0.040	1.25(1.01-1.56)
By cutoff					
D1 (< 296)	161/194		ref.		ref.
D2 (≥ 296)	82/50	0.001	1.98(1.32-2.99)	0.001	2.16(1.36-3.48)
Interquartile					
Q1~Q3 (< 305)	169/195		ref.		ref.
Q4 (≥ 305)	74/49	0.012	1.71(1.13-2.60)	0.024	1.74 (1.08-2.82)

Notes: The sensitivity analysis of the correlation between PLT and the one-year survival of the cancer cachexia population after excluding 19 cases who received preoperative treatment.

## Abbreviations

PLT: platelet count
ORs: odds ratios
95% CIs: 95% confidence intervals
OS: overall survival
SD: standard deviation
TBIL: total bilirubin
CHD: coronary heart disease
BMI: body mass index
MAC: mid-upper arm circumference
TSF: triceps skin fold
HGS: hand grip strength
AST: aspartic transaminase
ALT: alanine transaminase
WBC: white blood cell
RBC: red blood cell
Hb: hemoglobin
*P*: probability
D: dichotomy
Q: quarter
MPV: mean platelet volume
PDW: platelet distribution width
PCT: platelet crit
PLR: platelet-to-lymphocyte ratio
TNM: tumor node metastasis
